# Supplementary material for: Genetic Diversity and Expression of Intimin in Escherichia albertii Isolated from Humans, Animals, and Food
Source: Microorganisms. 2023 Nov 23;11(12):2843. doi: 10.3390/microorganisms11122843 (PMC10745426; doi:10.3390/microorganisms11122843)
Supplement: Supplementary file 1 [file microorganisms-11-02843-s001.zip › R1_Supplementary Figures.pdf]

Genetic Diversity and Expression of Intimin in *Escherichia albertii* Isolated from Humans, Animals, and Food

Qian Liu [1], Xi Yang [1], Hui Sun [1], Hua Wang [1], Xinxia Sui [1], Peihua Zhang [1], Xiangning Bai [1,2] and Yanwen Xiong [1,*]

[1] National Key Laboratory of Intelligent Tracking and Forecasting for Infectious Diseases, National Institute for Communicable Disease Control and Prevention, Chinese Center for Disease Control and Prevention, Beijing 102206, China

[2] Division of Laboratory Medicine, Oslo University Hospital, 0372 Oslo, Norway

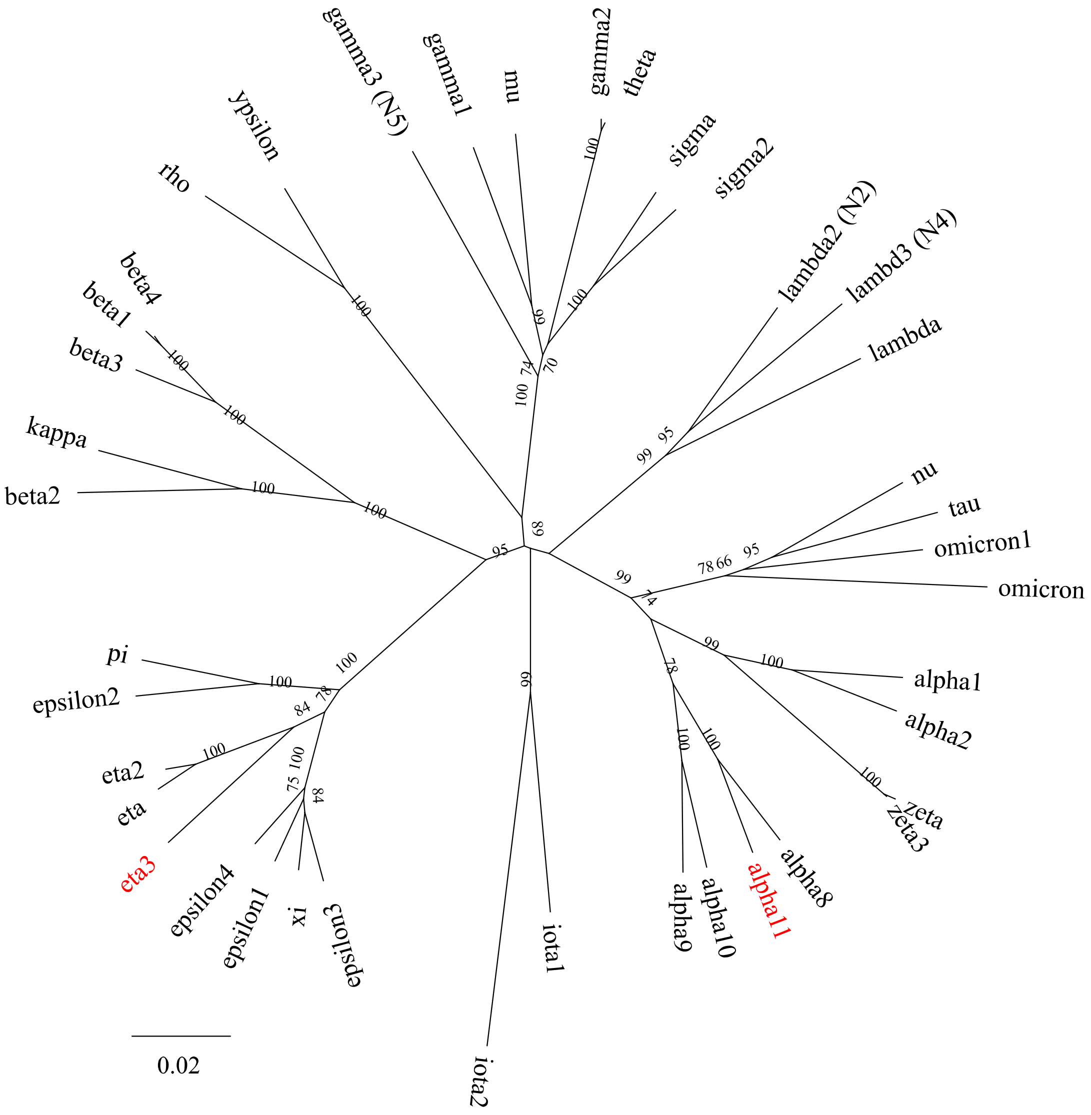

Supplementary Figure S1. Neighbor-joining (NJ) tree of 40 different subtypes of *eae* gene

Branches with a bootstrap of 50% support were labeled (1000 bootstrap, pairwise deletion). Two novel subtypes defined in this study were indicated in red.

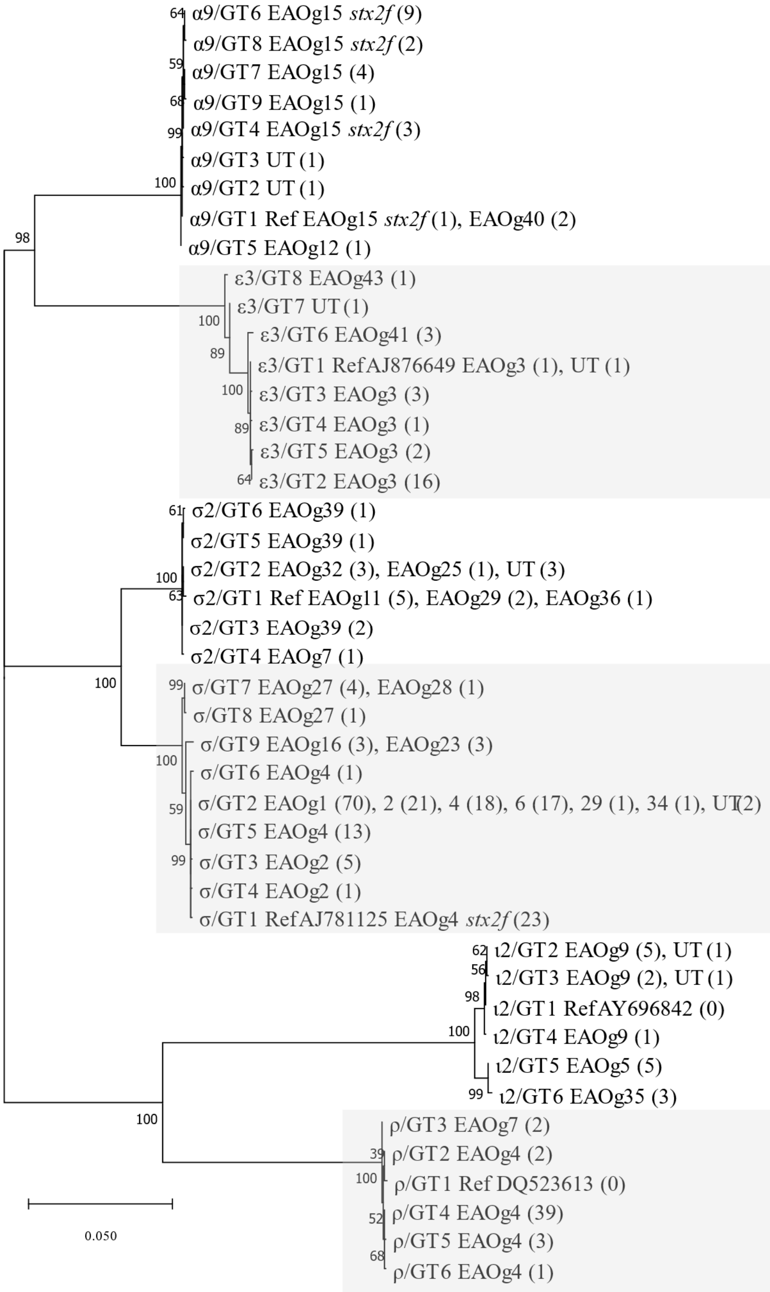

	Human	Animal	Food	Total
α9/GT6 EAOg15 *stx2f* (9)	1	8	0	9
α9/GT8 EAOg15 *stx2f* (2)	1	1	0	2
α9/GT7 EAOg15 (4)	0	4	0	4
α9/GT9 EAOg15 (1)	0	1	0	1
α9/GT4 EAOg15 *stx2f* (3)	0	3	0	3
α9/GT3 UT (1)	0	1	0	1
α9/GT2 UT (1)	0	1	0	1
α9/GT1 Ref EAOg15 *stx2f* (1), EAOg40 (2)	2	1	0	3
α9/GT5 EAOg12 (1)	1	0	0	1
ε3/GT8 EAOg43 (1)	0	1	0	1
ε3/GT7 UT (1)	0	1	0	1
ε3/GT6 EAOg41 (3)	0	3	0	3
ε3/GT1 RefAJ876649 EAOg3 (1), UT (1)	1	1	0	2
ε3/GT3 EAOg3 (3)	1	1	1	3
ε3/GT4 EAOg3 (1)	1	0	0	1
ε3/GT5 EAOg3 (2)	0	0	2	2
ε3/GT2 EAOg3 (16)	2	2	12	16
σ2/GT6 EAOg39 (1)	0	1	0	1
σ2/GT5 EAOg39 (1)	1	0	0	1
σ2/GT2 EAOg32 (3), EAOg25 (1), UT (3)	1	6	0	7
σ2/GT1 Ref EAOg11 (5), EAOg29 (2), EAOg36 (1)	1	7	0	8
σ2/GT3 EAOg39 (2)	1	1	0	2
σ2/GT4 EAOg7 (1)	0	1	0	1
σ/GT7 EAOg27 (4), EAOg28 (1)	5	0	0	5
σ/GT8 EAOg27 (1)	1	0	0	1
σ/GT9 EAOg16 (3), EAOg23 (3)	3	3	0	6
σ/GT6 EAOg4 (1)	0	1	0	1
σ/GT2 EAOg1 (70), 2 (21), 4 (18), 6 (17), 29 (1), 34 (1), UT (2)	31	15	85	131
σ/GT5 EAOg4 (13)	1	1	11	13
σ/GT3 EAOg2 (5)	0	0	5	5
σ/GT4 EAOg2 (1)	1	0	0	1
σ/GT1 RefAJ781125 EAOg4 *stx2f* (23)	1	22	0	23
ι2/GT2 EAOg9 (5), UT (1)	6	0	0	6
ι2/GT3 EAOg9 (2), UT (1)	3	0	0	3
ι2/GT1 RefAY696842 (0)	0	0	0	0
ι2/GT4 EAOg9 (1)	1	0	0	1
ι2/GT5 EAOg5 (5)	2	1	2	5
ι2/GT6 EAOg35 (3)	3	0	0	3
ρ/GT3 EAOg7 (2)	0	2	0	2
ρ/GT2 EAOg4 (2)	0	0	2	2
ρ/GT1 Ref DQ523613 (0)	0	0	0	0
ρ/GT4 EAOg4 (39)	8	0	31	39
ρ/GT5 EAOg4 (3)	0	0	3	3
ρ/GT6 EAOg4 (1)	1	0	0	1

Supplementary Figure S2. Phylogenetic relationships of 44 different *eae* sequences including 6 predominant *eae* subtypes in this study

The tree was based on the neighbor-joining method. The corresponding *eae* subtypes, genotypes (abbreviated as GT), and the number of strains isolated from different sources are listed on the right. The scale bar indicates genetic distance.

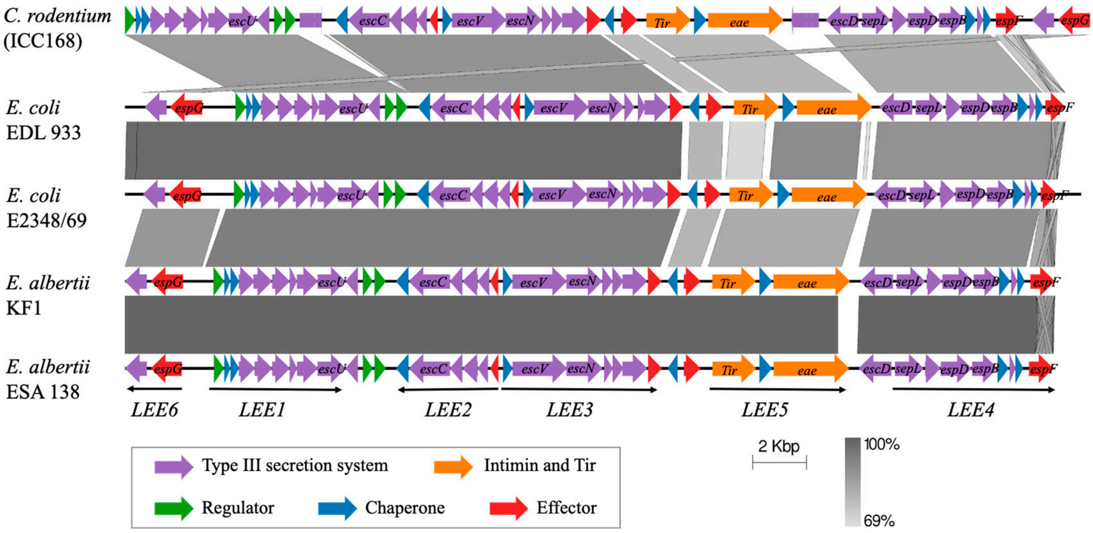

Supplementary Figure S3. The genetic structure of LEE

Four representative strains (*C. rodentium* strain ICC168, EHEC strain EDL933, EPEC strain E2348/69, and *E. albertii* strain NBRC 107761), and an *E. albertii* isolate (ESA138) were compared. Meta-alignment reveals that LEE is highly conserved between *E. albertii* and the other A/E members.
